# Relationship between olfaction and maxillofacial morphology in children with malocclusion

**DOI:** 10.1002/cre2.329

**Published:** 2020-09-25

**Authors:** Shiori Oka, Hitoshi Kawanabe, Shinya Yamanobe, Kazunori Fukui, Yuh Baba, Toru Deguchi

**Affiliations:** ^1^ Department of Oral Growth and Development, Division of Dentofacial Orthopedics Ohu University, Graduate School of Dentistry Fukushima Japan; ^2^ Division of Orthodontics and Dentofacial Orthopedics, Department of Oral Growth and Development Ohu University, School of Dentistry Fukushima Japan; ^3^ Department of General Clinical Medicine Ohu University, School of Dentistry Fukushima Japan; ^4^ Division of Orthodontics The Ohio State University College of Dentistry Columbus Ohio USA

**Keywords:** malocclusion, maxillofacial morphology, nasal air flow, olfaction

## Abstract

**Objectives:**

Functional problems, including nasal flow problems, are associated with specific skeletal and dental features. Further, maxillary expansion has been associated with nasal airway resistance alterations. This study aimed to investigate whether there is a correlation between skeletal features and nasal airflow‐ and olfaction‐related problems.

**Materials and methods:**

This prospective study included 68 patients (30 boys, 38 girls; mean age 9 ± 2 years) examined at the Ohu University Hospital. We classified patients into three skeletal Classes (Class I, II, and III) based on the ANB angle. Olfactory disorder history was collected from the guardians. Maxillofacial measurements, nasal airflow assessments, and olfactory tests were performed using cephalometric analysis, rhinomanometry, and T&T olfactometer, respectively.

**Results:**

Malocclusion, resulting from skeletal mandibular protrusion and smaller maxilla, was associated with reduced olfaction in children. The detection and recognition thresholds of skeletal Class III were significantly higher than those of Classes I (*p* = .01) and II (*p* = .01). Significant correlations were observed between SNA and the detection threshold (*r* = −.50) as well as between nasion perpendicular‐point A and the recognition threshold (*r* = −.53). The detection and recognition thresholds were significantly higher in Class III than in Classes I (*r* = .3) and II (*r* = −.1).

**Conclusions:**

Maxillary growth and development may be associated with olfaction in children. Changing the maxillofacial morphology may improve olfactory function. In the future, we will investigate how malocclusion treatment affects olfactory function.

## 1 INTRODUCTION

Functional problems associated with oral habits, mouth breathing, and tongue thrusts can be resolved using timely clinical orthodontic therapy (Zou, Meng, Law, Rao, & Zhou, 2018). However, if untreated, such problems can both affect skeletal characteristics in all three dimensions (anteroposterior [AP], vertical, and transverse) and influence dental characteristics. Mouth breathing in children has been particularly associated with palatal surface area and volume reduction, as well as altered palatal morphology (Lione et al., 2015). Sucking habits have been associated with the development of an anterior open bite and posterior crossbite (Dimberg et el., 2013; Odont, 2001). Moreover, sucking habits until the age of 5 years have been correlated with the presence of an atypical swallowing pattern at the age of 6–9 years, which in turn has been associated with the morphological severity score of malocclusion at the age of 12 years (Ovsenik, Farcnik, Korpar, & Verdenik, 2007). Prolonged mouth breathing may result in skeletal open bite with craniofacial and dental planes inclined in the posterior direction (Peltomäki, 2007).

Upper airway compromise may lead to chronic mouth breathing in dolichocephalic children (Hartgerink, Vig, & Abbott, 1987; Primožič, Franchi, Perinetti, Richmond, & Ovsenik, 2013). Furthermore, vertical dentofacial morphology may be related to airway problems (Kim, Hong, Hwang, & Park, 2010). However, several studies have focused on the relationship between sagittal (AP) dentofacial morphology and nasal airway problems (Bazargani, Magnuson, & Ludwig, 2018; Dimberg, Lennartsson, Söderfeldt, & Bondemark, 2013; Halicioğlu, Kiliç, Yavuz, & Aktan, 2010; Haralambidis et al., 2009; Iwasaki et al., 2013). Moreover, several studies have indicated that, with or without surgical treatment, skeletal maxillary expansion results in increased nasal space and improvement of the nasal airway (Guenthner, Sather, & Kern, 1984). Maxillary advancement is also suggested to result in the improvement of nasal airflow (Guenthner et al., 1984). Thus, morphological changes may impact the nasal airway, and specific skeletal features may be influenced by nasal airway problems.

Improvement in olfactory sensitivity may occur after maxillary expansion (Ottaviano et al., 2014). Smaller olfactory bulb volume is associated with higher olfactory thresholds (Hummel, Haehner, Hummel, Croy, & Iannilli, 2013). Interestingly, improvement in olfactory thresholds was observed after rapid maxillary expansion in a pilot study (Ottaviano et al., 2014). Enlargement of the upper jaw bone has been shown to lead to changes in the lateral volume of the nasal cavity, thereby improving nasal congestion and nasal permeability and reducing the olfactory threshold. Impaired olfactory function could be life‐threatening, because it may lead to consumption of spoiled food as well as unawareness of fires and gas leaks. If orthodontic treatment can improve olfactory function, it may be beneficial to widen the maxilla by treatment during childhood. Thus, we investigated whether a direct relationship exists between olfactory sensitivity (and nasal airway flow) and skeletal morphology.

The objective of this study was to investigate the relationship between skeletal features and the occurrence of problems related to nasal airflow and olfaction. If a specific skeletal feature causes olfactory problems, changing the morphology of the maxillofacial complex may achieve improved olfaction.

## 2 MATERIALS AND METHODS

### 2.1 Study sample

This study was approved by the Ethics Screening Committee of Ohu University, Koriyama, Japan (Approval No. 139) and was conducted in accordance with the Declaration of Helsinki. Informed consent was obtained from the participants and their guardians. Subjects in this prospective study included 68 patients (30 boys and 38 girls) with a mean ± standard deviation (*SD*) age of 9 ± 2 years (range: 6–12 years), who were examined at the Department of Orthodontics at Ohu University Hospital. The values for mean and *SD* age are presented as integers.

Children with nasal or congenital diseases were excluded from participation. Furthermore, histories of olfactory disorders in the children were collected from their guardians (Kobayashi, 2014). All patients examined at the Department of Orthodontics at Ohu University Hospital during the ethics approval period, and who did not have the exclusion criteria, were considered for participation.

### 2.2 Maxillofacial morphology

Based on the skeletal malocclusion classification described by McNamara (1984), there were 22, 24, and 22 patients in skeletal Classes I, II, and III, respectively (McNamara, 1984; Proffit, Fields, & Sarver, 2013). Moreover, anthropometric characteristics were measured. Maxillofacial morphology was assessed by one investigator (SO), who measured models of the oral cavity, traced lateral cephalograms, and used software to analyze the cephalometric data (Dolphin imaging software version 11.9, Dolphin Imaging Systems, LLC, Chatsworth, CA) and designate measurement points according to the method described by McNamara (1984).

### 2.3 Rhinomanometry

Rhinomanometry was performed using a multifunctional spirometer (HI‐801, CHEST M.I. Inc, Tokyo, Japan) in accordance with the rhinomanometry guidelines of the Japanese Rhinologic Society (Naito, Miyazaki, & Nonaka, 2001). Rhinometry is a method that simultaneously measures the air flow velocity and the pressure difference before and after the nasal cavity during spontaneous breathing at rest (Naito et al., 2001). Specifically, anterior rhinomanometry is easy to perform, does not cause significant patient discomfort, and can be performed quickly. However, it cannot be used in patients with unilateral nasal cavity obstruction. The International Committee on Rhinomanometry Standards has defined the standard resistance value as Δ*P* 150 Pa. However, in many Japanese individuals with normal nasal cavities, a resistance of 150 Pa cannot be achieved during resting respiration; therefore, the Japanese Rhinomanometry Committee recommends a resistance of 100 Pa. Anterior rhinomanometry was performed on both left and right sides. Before measurements, patients were instructed to rest in a sitting position with the head in a natural position. Measurements during inspiration were used in this study. Bilateral nasal cavity resistance was calculated from the right and left resistance values using the Ohm's law equation. Nasal cavity resistance was expressed as Pa/cm^3^/s.

### 2.4 Olfactory test

For the olfactory test, we used a T&T olfactometer (Daiichi Yakuhin Sangyo, Tokyo, Japan) and followed a previously described method to perform standard olfactory measurements. Specific reagents were used in accordance with the manufacturer's recommendations (Daiichi Yakuhin Sangyo Ltd., Tokyo, Japan) (Oka et al., 2013; Takagi, 1978). The following scents were used to measure detection and recognition thresholds: A, β‐phenylethyl alcohol; B, methyl cyclopentenolone; C, *iso*‐valeric acid; D, γ‐undecalactone; and E, skatole. The detection threshold was the concentration, at which a scent was sensed. The recognition threshold was determined by further increasing the concentration until the type of scent was identified. The test was single‐blinded; each test was performed only once due to the possibility of olfactory fatigue.

Olfaction measurements involved increasing the concentration of the standard scents from low to high (−2 to +5); +5 represented the highest concentration and strongest scent. Each successive concentration strength increased the scent by a factor of 10. When the patient perceived the standard scent, the corresponding concentration was recorded on an olfactogram (detection threshold). The concentration was then increased further, and when the patient could correctly identify the type of standard scent, it was recorded on the olfactogram (recognition threshold). Patients with T&T recognition thresholds 5.5 or higher were diagnosed with anosmia. Furthermore, patients were divided into five Classes based on their olfactory function (Table 1).

**TABLE 1:** Description of standard odors^a^ and categorization of olfactory sensitivity into five Classes based on mean recognition thresholds in olfactograms^a^

Standard odors	Qualities of standard odors
A	Odor of rose or light sweet odor
B	Burnt odor or caramel odor
C	Putrid odor, odor of long‐worn socks, sweaty odor, or odor of fermented soybeans
D	Canned peach odor or heavy sweet odor
E	Fecal odor, odors of vegetable garbage, oral odor, or aversive/bad odor

Olfactory loss: Average recognition threshold	Degree of olfactory disorder	Complaint of patient
~1.0	Normal or normosmic	All odors detected normally No olfactory problem in daily life
1.1 ~ 2.5	Normal or mildly hyposmic	All odors detected mildly
2.6 ~ 4.0	Moderately hyposmic	Only strong odors detected
4.1 ~ 5.5	Severely hyposmic	Odors scarcely detected
5.5~	Anosmic	No odors detected

^a^ Courtesy of Igaku‐Shoin, Ltd., Tokyo.

Mean detection and recognition threshold values for scents A to E were calculated based on the detection and recognition thresholds recorded on olfactograms in the above measurements. Olfaction was assessed as normal or reduced and categorized into 5 grades based on the criteria listed in Table 1. The mean recognition threshold was adopted for the assessments.

### 2.5 Statistical analyzes

Statistical analyzes were performed using SPSS version 24.0 (IBM Corporation, Armonk, NY). Comparisons of maxillofacial morphology, olfaction, oral cavity models, and rhinomanometry among skeletal Class I, II, and III malocclusion groups were performed using the Kruskal–Wallis test. Multiple comparisons of these results were performed utilizing the Mann–Whitney *U* test. Correlations between maxillofacial morphology and olfaction were analyzed using Spearman's rank correlation coefficient; *p* < .05 was considered statistically significant.

## 3 RESULTS

### 3.1 Patient characteristics

There were no significant differences between boys and girls in terms of maxillofacial morphology, nasal cavity air flow, and olfactory function at the initial examination, including Rohrer index (*p* < .05) (Table 2).

**TABLE 2:** Patient characteristics of skeletal Class I, II, and III groups

	Skeletal Class I	Skeletal Class II	Skeletal Class III	Kruskal–Wallis
	(*n* = 22)	(*n* = 24)	(*n* = 22)	
Height (cm) (±*SD*)	134.39 (± 10.81)	134.82 (± 9.12)	134.40 (± 9.06)	NS
Weight (kg) (±*SD*)	32.30 (± 10.77)	30.76 (± 6.99)	30.10 (± 5.34)	NS
BMI (±*SD*)	17.43 (± 3.43)	16.79 (± 2.58)	16.60 (± 1.52)	NS
Rohrer index (±*SD*)	129.65 (± 21.03)	109.00 (± 12.00)	123.80 (± 12.80)	NS

Abbreviation: NS, not significant.

### 3.2 Maxillofacial morphology

Table 3 summarizes the results of Kruskal–Wallis tests among the three skeletal groups with regards to measurements from lateral cephalograms. SNB, McNamara to Pogonion, facial angle, and Frankfurt mandibular plane angle (U1‐FH) were significantly smaller in skeletal Class II than in skeletal Class I (*p* < .05). ANB and Frankfurt mandibular plane angle were significantly larger in skeletal Class II (*p* < .05) (Table 3).

**TABLE 3:** Comparison of cephalometric measurements among patients

	Skeletal	Skeletal	Skeletal	Kruskal–Wallis I vs II	Kruskal–Wallis I vs III	Kruskal–Wallis II vs III
	Class I	Class II	Class III			
	(n = 22)	(n = 24)	(n = 22)			
	*M* (±*SD*)	*M* (±*SD*)	*M* (±*SD*)			
SNA (°)	80.54 (± 2.83)	80.24 (± 3.83)	79.53 (± 3.01)	NS	*	*
SNB (°)	77.42 (± 2.76)	73.98 (± 3.66)	80.10 (± 3.35)	**	NS	**
ANB (°)	3.12 (± 0.54)	6.24 (± 1.92)	−0.10 (± 1.18)	**	**	**
Nasion perpendicular‐point A (mm)	−1.04 (± 3.38)	−0.01 (± 3.13)	−2.40 (± 2.40)	NS	*	**
McNamara to Pogonion (mm)	−8.38 (± 6.49)	−11.47 (± 6.84)	−4.30 (± 4.41)	*	NS	**
Facial A. (°)	85.89 (± 3.06)	83.80 (± 3.74)	87.40 (± 2.99)	*	*	*
FMA (°)	28.86 (± 4.33)	31.63 (± 5.72)	28.90 (± 5.07)	*	NS	NS
U1‐FH (°)	118.43 (± 7.48)	112.24 (± 5.91)	112.50 (± 6.14)	*	*	NS
L1‐Mp (°)	88.85 (± 15.81)	94.84 (± 5.54)	85.30 (± 5.49)	NS	*	**
Overbite (mm)	2.33 (± 2.24)	3.10 (± 1.64)	2.50 (± 2.10)	NS	NS	NS
Overjet (mm)	3.88 (± 2.88)	4.27 (± 2.12)	−0.6 (± 2.76)	NS	*	**

Abbreviation: NS: not significant.

^*^ *p* < .05.

^**^ *p* < .01.

The facial angle was significantly larger in skeletal Class III than in skeletal Class I (*p* < .05). SNA, ANB, and nasion perpendicular‐point A were significantly smaller in skeletal Class III (*p* < .05). U1‐FH, L1 to mandibular plane angle, and overjet were significantly smaller in skeletal Class III (*p* < .05) (Table 3).

SNA, ANB, nasion perpendicular‐point A, L1 to mandibular plane angle, and overjet were significantly smaller in skeletal Class III than in skeletal Class II (*p* < .05). SNB, McNamara to Pogonion, and facial angle were significantly larger in skeletal Class III (*p* < .05) (Table 3).

Maxillary basal arch widths and mandibular basal arch widths and lengths were not significantly different between skeletal Classes I and II, skeletal Classes I and III, or skeletal Classes II and III (*p* < .05). However, maxillary basal arch lengths were significantly smaller in skeletal Class III than in skeletal Classes I and II (*p* < .05).

### 3.3 Maxillofacial morphology and olfaction

**FIGURE 1:**
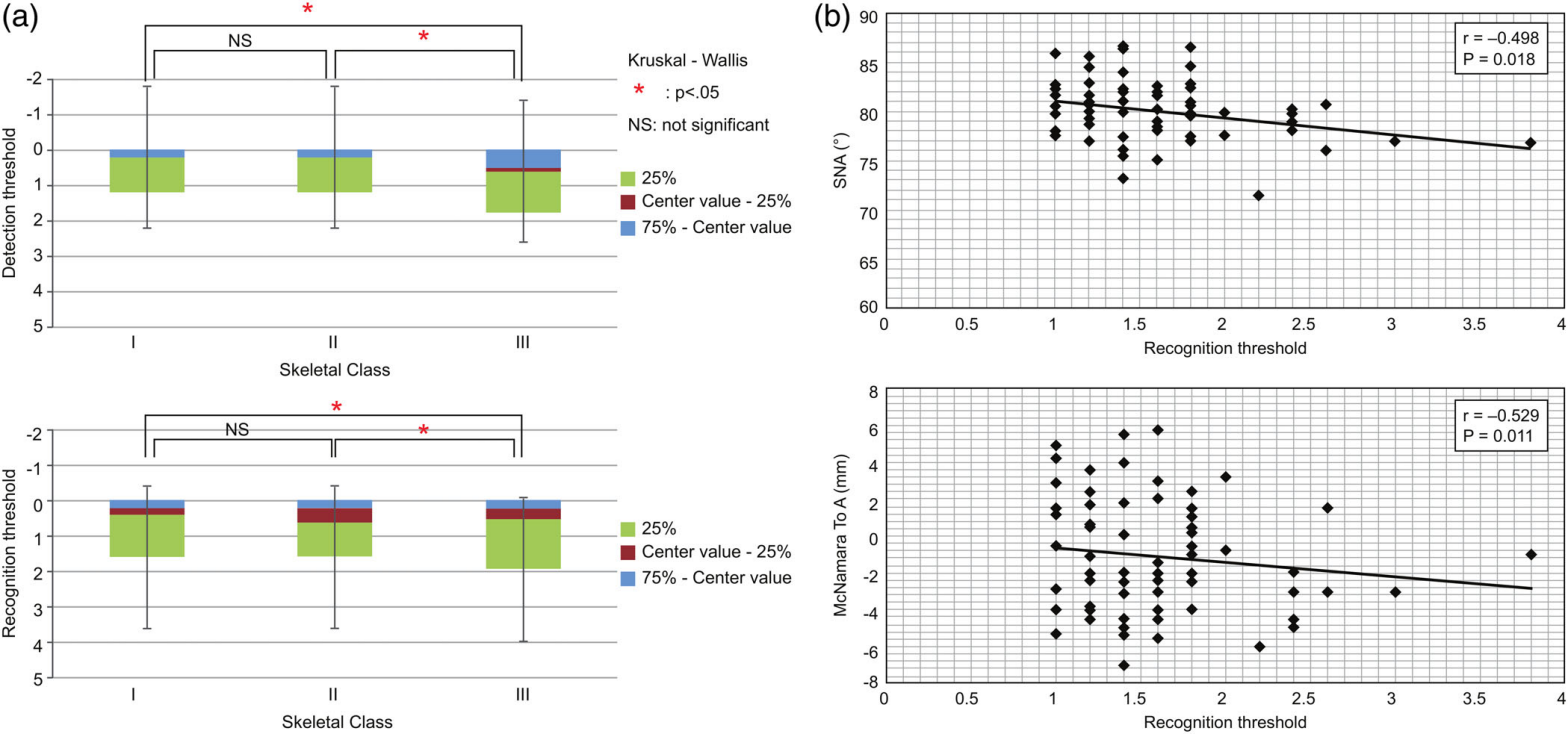
Relationship between skeletal changes and olfaction. (a) Correlation between maxillofacial morphology and olfaction. (b) Relationship between maxillofacial morphology and olfaction

No significant differences in the detection and recognition thresholds were observed between skeletal Classes I and II (*p* < .05). However, the detection and recognition thresholds of skeletal Class III were significantly higher than those of skeletal Classes I and II (*p* < .05) (Figure 1a).

### 3.4 Correlations between maxillofacial morphology and olfaction

Significant correlations were observed between SNA and the detection threshold (*r* = −.50) and between nasion perpendicular‐point A and recognition threshold (*r* = −.53). No significant correlations in detection or recognition thresholds were observed between skeletal Classes I and II. However, the detection and recognition thresholds were significantly higher in skeletal Class III than in skeletal Classes I (*p* = .01) and II (*p* = .01) (Figure 1b).

### 3.5 Maxillofacial morphology and nasal cavity air flow

No significant differences were observed between any maxillofacial morphological parameters and nasal cavity resistance (*p* < .05).

## 4 DISCUSSION

Here, we investigated morphological characteristics in different AP relationships, because specific functional problems may influence skeletal and dental aspects in normal patients. To determine whether our patients represented typical skeletal Class I, II, or III malocclusion in Japanese children, we compared mean height, weight, body mass index, and Rohrer index values with data from a survey of school dental health statistics (Lifelong Learning Policy Bureau, Policy Department, Survey and Statistics Planning Office, 2016); we found the values to be similar in both cases. In our analysis, skeletal Class II patients tended to have a retruded chin, high angle, and flared maxillary incisors. This is consistent with previous reports that patients with functional problems, such as tongue thrusting and thumb sucking, often have a skeletal Class II relationship. In contrast, Class III patients have a protrusive chin, reduced axial inclination of incisors, and a small maxilla. Several studies have reported a higher incidence of pediatric obstructive sleep apnea syndrome (OSAS) among patients with poor maxillary growth (Zhong, Tang, Gao, & Zeng, 2010). Thus, as underdevelopment of the maxilla is observed in Class III patients, a functional problem related to the sinus or nasal airway may be observed more frequently in Class III patients.

We investigated the relationship between morphological aspects and nasal cavity airway flow. A past study indicated that nasal cavity air flow was affected in OSAS children with malocclusion and poor maxillary growth (Iwasaki, 2016). The investigators reported that Class II patients had higher resistance than Class III patients. This finding contrasts with our results (i.e., lower nasal cavity airway flow in Class II patients). In the previous report, adenoid and tonsil hypertrophy patients were included; in our study, we excluded patients with functional problems. Subjects in the present investigation were children; children tend to have higher nasal cavity resistance than adults, which declines as they mature (Kobayashi et al., 2012; Koh et al., 2011). Previously, the mean nasal resistance of Japanese children has been reported as 0.43 ± 0.50 Pa/cm^3^/s (Kobayashi et al., 2012). In the present study, the nasal resistances were 0.50 ± 0.30 Pa/cm^3^/s, 0.45 ± 0.10 Pa/cm^3^/s, and 0.50 ± 0.25 Pa/cm^3^/s in skeletal Classes I, II, and III, respectively; these results are consistent with those of earlier studies (Kobayashi et al., 2011, 2012). Moreover, nasal cavity resistance did not differ significantly among the three groups. Therefore, maxillofacial morphology may not be associated with differences in nasal cavity air flow.

Mori et al. (2011) reported that 41, 14, and 7% of pediatric medical institutions, hospitals, and clinics, respectively, which constitute less than 50% of pediatric institutions, perform standard olfactory tests. The T&T olfactometer test requires specialized equipment as well as time and effort. Hashimoto et al. (2004) compared the results of the T&T olfactometer and the cross‐cultural smell identification test and revealed a significant correlation with clinical outcomes. Fujii, Fukazawa, and Sakagami (2001) showed that the results from a T&T olfactometer correlated significantly with the results from the odor stick identification test. Thus, we selected the T&T olfactometer to measure olfaction, because it is simple to use and is covered by health insurance.

In this study, a reduction in olfactory function was observed in skeletal Class III patients. Sorokowska et al. (2015) have reported that olfaction matures by approximately 10 years of age and changes very little until the sixth decade of life. Scammon growth curves indicated that 90% of neural growth and development occurs by 6 years of age and is complete by 20 years of age. Subjects in the present study were 6–12 years of age. Improving olfaction early by normalizing olfactory threshold values could enhance the quality of life.

Hummel et al. (2013) have reported that differences in olfactory bulb volumes are associated with lateralized differences in olfactory function in humans. They observed that a larger olfactory bulb volume correlated with improved olfactory function and greater sensitivity. Moreover, different olfactory bulb volumes on the left and right may be related to asymmetric olfactory capability. Furthermore, Altundag et al. (2014) studied lateralized differences in olfactory function in patients with nasal septum deviation and reported that the olfactory threshold and the differentiation and identification of the type of smell were lower (less sensitive) on the narrower side. Moreover, significant positive correlations were observed between the olfactory bulb volume and the olfactory threshold, and between the differentiation and identification of the type of smell.

In the present study, we did not examine olfactory bulb volume in the study participants. However, De Felippe, Bhushan, Da Silveira, Viana, and Smith (2009) have reported that upper airway resistance decreases with the increasing nasal cavity volume by rapid maxillary expansion. Moreover, Cappellette, Alves, Nagai, Fujita, and Pignatari (2017) have found that rapid maxillary expansion expands all structures of the nasomaxillary complex (nasal cavity, oropharynx, and left and right maxillary sinuses). Ottaviano et al. (2014) have suggested that rapid maxillary expansion improves peak nasal inspiratory flow, reduced nasal resistance, and improved olfaction thresholds. Thus, treatment of malocclusion in children with skeletal mandibular protrusion and small maxilla may improve olfaction.

## 5 CONCLUSION

Children with malocclusion due to skeletal mandibular protrusion and poor maxillary growth tended to exhibit reduced olfaction, which suggests a correlation between olfaction and maxillary growth and development. Further studies are needed to investigate whether malocclusion treatment impacts olfactory function.

## CONFLICT OF INTEREST

The authors declare no conflict of interest.

## Data Availability

The data that support the findings of this study are available from the corresponding author upon reasonable request.
